# Investigating the research landscape of chlorinated paraffins over the past ten decades

**DOI:** 10.3389/ftox.2024.1533722

**Published:** 2025-01-22

**Authors:** Chinemerem Ruth Ohoro, Chijioke Olisah, Victor Wepener

**Affiliations:** ^1^ Water Research Group, Unit for Environmental Sciences and Management, North-West University, Potchefstroom, South Africa; ^2^ Institute for Coastal and Marine Research (CMR), Nelson Mandela University, Gqeberha, South Africa

**Keywords:** persistent organic pollutants (POPs), chlorinated paraffin, scientometric analysis, short-chain chlorinated paraffin, medium-chain chlorinated paraffin, long-chain chlorinated paraffins

## Abstract

Chlorinated paraffins (CPs) are classified as emerging persistent organic pollutants (POPs). Due to their associated environmental and health impacts, these groups of chemicals have been a subject of interest among researchers in the past decades. Here we used a scientometric approach to understand the research landscape of CPs using literature published in the Web of Science and Scopus database. RStudio and VOSviewer programs were employed as scientometric tools to analyze the publication trends in global CP-related research from 1916 to 2024. A total of 1,452 articles were published over this period, with a publication/author and co-author/publication ratio of 0.43 and 5.49, respectively. China ranked first in publication output (n = 556, 43.3%), and the highest total citations (n = 12,007), followed by Sweden (n = 90), Canada (n = 77), and Germany (n = 75). Publications from developing countries were limited, with most contributions from Africa originating from Egypt (n = 7), South Africa (n = 5), and Nigeria (n = 3), primarily through international collaborations. The average annual growth rate of 4.3% suggests a significant future article output. This scientometric analysis allowed us to infer global trends in CPs, identify tendencies and gaps, and contribute to future research. Despite having similar toxicity to short-chain chlorinated paraffin (SCCP), long-chain chlorinated paraffin (LCCP) has received less attention. Therefore, future research should prioritize studying LCCP bioaccumulation and toxicity in diverse food webs, focusing on aquatic species vulnerable to CPs and effective toxicological models. Additionally, collaborative research with developing countries should be encouraged to enhance meeting the Stockholm Convention’s demand.

## 1 Introduction

Persistent organic pollutants (POPs) might remain near their sources or travel long-range in the atmosphere or oceans. Therefore, emissions from one country can contaminate another country (Jones, 2021; Ohoro et al., 2022); consequently, they are regulated globally (Gong et al., 2021). Due to their inability to biodegrade, they may persist in the environment for long. They can also bioaccumulate in plants, aquatic organisms, and humans, where they can become potentially harmful (Aravind Kumar et al., 2022). Chlorinated paraffins (CPs), categorized as emerging POPs (Huang et al., 2023) are one of the most prevalent challenges in current analytical chemistry (Fernandes et al., 2022a). Due to their widespread use, persistence, and long-range atmospheric transport, they are prevalent in the environment, particularly in the oceans where they are transformed (Lyu and Zhang, 2023). For ages, these anthropogenic compounds have been extensively utilized in the manufacturing of rubber, textiles, leather goods, pigments, sealing agents, adhesives, plasticizers, fluids for welding, lubricant additives, thermal regulators for metalworking, and flame retardants (Huang et al., 2023; Fernandes et al., 2022a; Vetter et al., 2022; Chen C. et al., 2022; Yuan S. et al., 2021; Guida et al., 2020; Mu et al., 2023).

An estimated 1,000,000 tonnes of CPs are produced annually (Mézière et al., 2021), with China being the major producer (Pan et al., 2021), followed by India (Guida et al., 2020). Carbon-chain length categorizes CPs into three groups: short-chain (SCCP, C_10–13_), medium-chain (MCCP, C_14–17_), and long-chain (LCCP, C_18–30_) (Mu et al., 2023; Chen et al., 2023). Sources of CPs include the leaching and release of marine plastic litter (Lyu and Zhang, 2023), discharges from manufacturing and consumption (Xia et al., 2021), polyvinyl chloride curtains, ovens, and window glass (Beloki Ezker et al., 2024). The two main ways of exposure to CPs are by ingestion and inhalation of contaminated dust and air (Yuan B. et al., 2021b), others include dermal exposure (Liao et al., 2023; Gao et al., 2021; Zhu et al., 2024; Yuan et al., 2022a), nail and hair contact, breastfeeding (Mu et al., 2023; Chain et al., 2020), and exposure during pregnancy (Chen et al., 2023). CPs may result in diabetes, asthma, immunological dysfunction, endocrine disruptions, neurotoxicity, developmental toxicity, liver and kidney toxicity, mutagenicity, carcinogenicity, and reproductive toxicity (Huang et al., 2023; Mu et al., 2023; Yuan B. et al., 2021; Liao et al., 2023; Zhu et al., 2024; Tahir et al., 2024a). They have been detected in various matrices such as water and sediment (Guan et al., 2023; Tahir et al., 2024b), plants (Wang et al., 2021), seawater (Hu et al., 2022), groundwater (Wu et al., 2021), municipal solid waste incineration (Han et al., 2024), soil and sediment (Wu et al., 2020; Wang K. et al., 2020), foodstuff (Ding et al., 2021; Lee et al., 2020; Cui et al., 2020; Perkons et al., 2022; Han et al., 2021; Li et al., 2020), biota samples (Yuan et al., 2022b; Wang W. et al., 2020; Girones et al., 2023; Lee et al., 2022; Tomasko et al., 2021), cow milk and feed (Dong et al., 2020), serum (Ding et al., 2020), human blood (Niu et al., 2023), breastmilk (Krätschmer et al., 2021; Xu et al., 2021; Wang et al., 2024; Zhou et al., 2020), placenta (Liu et al., 2020), and toys (Zhang et al., 2023). From this we can see that CPs have been widely studied; hence there is a need to investigate the research progression over the years.

Despite having the same toxicity as SCCP, MCCP, and LCCP were used as substitutes but not as well-studied (Ding et al., 2020; McGrath et al., 2021; He et al., 2023; South et al., 2022; McGrath et al., 2022); and might not be a reliable replacement for SCCPs, leaving gaps in safety assurance (Chen et al., 2023). Owing to their growing health concern, SCCPs were added to the Stockholm Convention’s POPs list in 2017, and recently, MCCPs have been proposed for inclusion as POPs under the Stockholm Convention, with the formal decision anticipated in 2025 (UK Government Department of Environment Food and Rural Affairs, 2024). While MCCPs and LCCPs receive less attention and have limited regulations in place for their management, SCCPs are extensively studied and regulated (Huang et al., 2023; Liao et al., 2023; Zhou et al., 2020; Jain et al., 2022).

SCCPs and MCCPs have been widely studied due to their growing industrial use and environmental and health implications. Given the consistent research outputs and regulation of this pollutant category under the Stockholm Convention on POPs, a bibliometric review of global research on CPs is necessary. Such review can map global publication trends, inform policy, foster research collaboration by highlighting networks and funding sources, and guide future studies, particularly around LCCPs that have been understudied. Their limited research could be due to analytical difficulties, such as the requirement for advanced detection techniques, appropriate standards, and low volatility that limits their atmospheric dispersion (South et al., 2022); as a result, exposure risk may be less of a concern. Research funding and attention are being discouraged because there is a limit to global regulations addressing their rising emissions, particularly their persistence, bioaccumulation, and possible toxicity in the marine environment (Lyu and Zhang, 2023). Their low solubility and high molecular size can also reduce their uptake and absorption by aquatic organisms (Adetunji et al., 2022); consequently, less motivation to investigate their occurrences in aquatic environments.

A bibliometric analysis is a branch of scientometrics that employs statistical methods to evaluate scientific output, identifying trends, key contributors, and knowledge gaps while assessing research productivity (Olisah et al., 2024; Joshi, 2016; Aria and Cuccurullo, 2017). This evaluation tracks research trends, publication impact, and key topics (Siamaki et al., 2014; Tomy and Boer, 2010), using indicators such as annual scientific production, most cited articles, collaboration networks, total citations per country, most productive authors, most cited authors, most relevant sources, and most relevant keywords. To date, no scientometric study on CPs has been conducted based on available information. Therefore, this study presents the first scientometric evaluation of CP research over the past century, providing insights into the field’s evolution and major themes. It aims to analyze global research on CPs from January 1916 to October 2024 using bibliometric tools to analyze articles indexed in WoS and Scopus, and addressing seven key research questions (1): What are the research outputs on CPs in ecosystems before and after the ban? (2) How has collaboration influenced CP research output? (3) What are the citation and authorship patterns? (4) What are the publication trends over the past 10 decades? (5) What thematic areas related to CPs have been most studied? (6) Are these themes potential drivers for future research? (7) Which CP classes have drawn the most research attention? This study helps researchers identify gaps and opportunities in the field.

## 2 Methodology

### 2.1 Data collection and inclusion criteria

**FIGURE 1:**
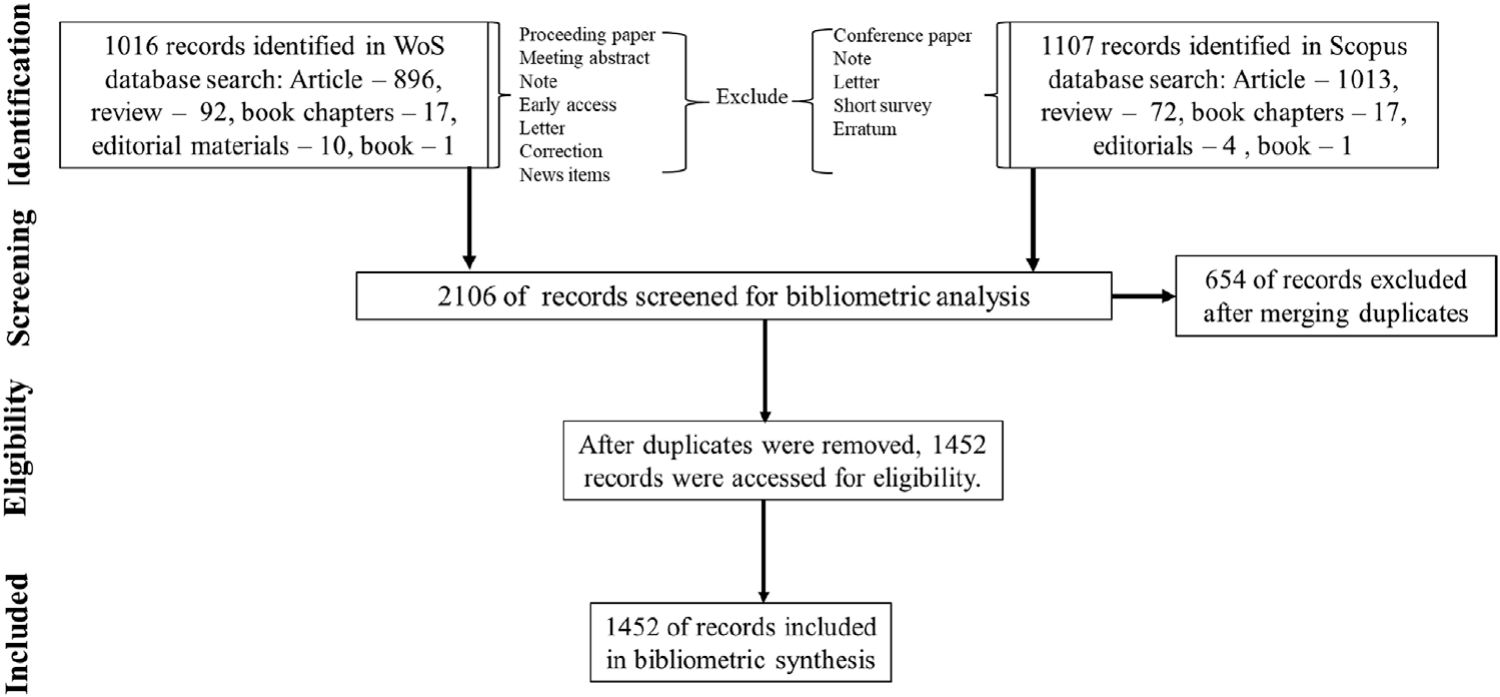
Selection criteria for publications included in the bibliometric analysis of chlorinated paraffins from the Web of Science and Scopus databases.

In this bibliometric study on chlorinated paraffins (CPs), we collected and analyzed publications from two major academic databases: the Web of Science Core Collection and Scopus. The Web of Science was chosen for this study because of its rich dataset of physical and biological science articles. The Scopus database, launched by Elsevier in 2004, was also selected for its comprehensive coverage of literature, enriched citation metrics, and detailed abstract sources (Reth and Oehme, 2004). The data collection aimed to provide a comprehensive understanding of the research landscape on CPs by examining publication patterns, document types, and research trends over time. The search term “chlorinated paraffin*” was used in the Web of Science Core Collection topic and the “Article Title, Abstract, Keyword” fields in Scopus. The search spanned all available years in each database, covering publications from 1 January 1990, to 4 October 2024, in Web of Science, and from 1 January 1916, to 4 October 2024, in Scopus. An initial search in the Web of Science Core Collection yielded a total of 1,070 documents related to CPs. These documents were categorized into several types: articles, review articles, proceeding papers, book chapters, meeting abstracts, editorial materials, notes, early access items, letters, corrections, news items, and books (Figure 1). To maintain a focus on peer-reviewed and substantial academic content, we excluded certain document types from the analysis. Excluded items included proceeding papers, meeting abstracts, notes, early access items, letters, corrections, and news items, resulting in a refined dataset of 1,016 Web of Science Core Collection documents. Similarly, a search in Scopus yielded 1,196 documents on CPs, which were also categorized by type: articles, review articles, conference papers, book chapters, notes, editorials, erratum, letters, short surveys, and books (Figure 1). Consistent with the criteria applied to the Web of Science dataset, certain document types were excluded from the Scopus dataset, including conference papers, notes, erratum, letters, and short surveys. This resulted in a total of 1,107 documents from Scopus. After excluding duplicate entries and merging the results from both databases using R (Version 1 April 1,106 ^©^ 2009–2021 RStudio, PBC), a total of 1,452 unique documents were identified and used for the bibliometric analysis.

### 2.2 Scientometric analysis

This final dataset provided a robust foundation for evaluating trends in CP research, allowing for a more detailed exploration of publication patterns, research focus, and key contributors within the field. Data from both databases were imported into RStudio (Version 1 April 1,106 ^©^ 2009–2021 RStudio, PBC) for bibliometric analysis. Key bibliometric indicators analyzed included the number of documents and authors, annual scientific productivity, top authors by productivity, most-cited manuscripts, corresponding authors’ countries, total citations by country, most relevant journals, and frequently used keywords. Codes with slight modifications used to analyze these indicators were adopted from Aria and Cuccurullo (Krätschmer and Schächtele, 2019). Additionally, thematic area classification and the country collaboration network were analyzed using VOSviewer (version 1.6.15 ^©^ 2009–2022).

## 3 Results and discussions

### 3.1 Literature demographics

**FIGURE 2:**
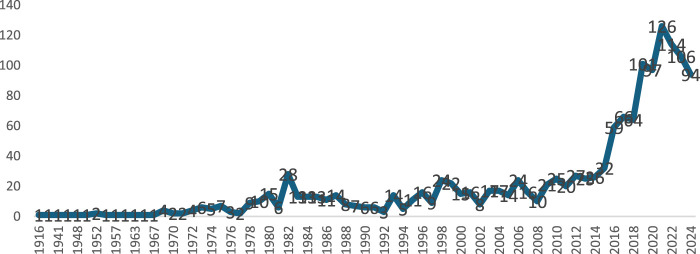
The annual publication frequencies on CPs globally published between 1916 and 2024. The annual growth rate was 4.3%.

This study analyzed 1,452 documents on CPs retrieved from the WoS and Scopus databases, covering January 1916 to October 2024. Articles made up most publications, accounting for 89.7% (n = 1,299), followed by Reviews (7.5%, n = 108), Editorials (0.1%, n = 2), and Book Chapters (0.8%, n = 12). Only 113 were single-authored papers, with ratios of 0.43 publications/author, and 5.49 co-authors/publication. The citation/publications and citation/year averages for these documents were 2.86 and 25.05, respectively. A high level of co-authorship participation is revealed by the study’s collaboration index, which is roughly 17.49 per article (van Mourik et al., 2016). About 3,353 authors contributed to the literature, with 83 authors producing single-authored works and 7,968 author appearances, retrieved from 443 journal and book sources. The publication output was low from 1916 to 1977 (Figure 2), likely due to the analytical challenges posed by CPs. The extreme complexity of CP mixtures, comprising thousands of stereoisomers, presents substantial difficulties for gas chromatography separation, hindering congener-specific analysis and limiting research on CP composition in environmental samples (Guida et al., 2020; Fiedler and Boer, 2010; Vorkamp et al., 2019; Glüge et al., 2016; Feo et al., 2009). There was a rapid increase in 1982 which could be a result of the 1980s production boom spurred on by the plastics and paint industry’s high demand for the use of SCCP as additives (Chen C. et al., 2022; Lipnick and Muir, 2000; Chen L. et al., 2022). This could also be similar to the rapid increase in Europe and Japan in 1977 (Alipour Parvizian et al., 2024), having growing concern due to the awareness of the widespread persistence, bioaccumulation, toxicity, and vast industrial use (UNEP, 2017; UNEP, 2015). Consequently, toxicological information regarding MCCP and LCCP was generated during this time (Liao et al., 2023), and biotransformation research was conducted (Arko et al., 2024). The output of articles decreased after that, but it increased again in 2015 (n = 32), and it has remained as such since then. There has been an upsurge in publications related to CPs (n = 768, 53%), since SCCPs were listed in the Stockholm Convention (2017–2024), compared to 235 articles generated before the listing (2009–2016).

### 3.2 Geographical distribution of publications and collaboration patterns

**FIGURE 3:**
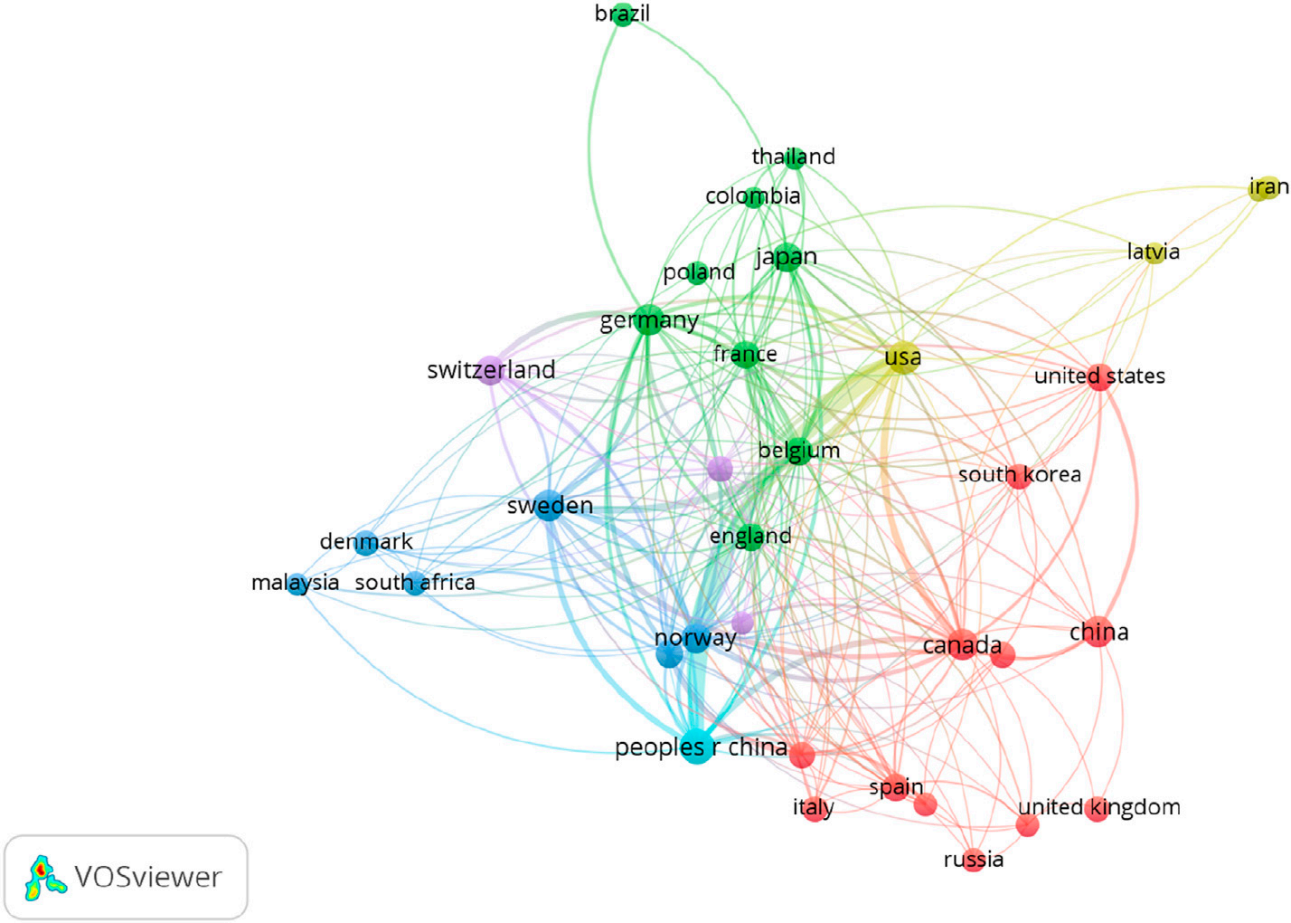
Collaboration pathways of countries involved in CP research across the globe.

Table 1 lists the top 25 corresponding author countries and citations for research regarding CPs conducted globally between 1916 and 2024, directed via publication and citation indices. The four countries with the highest publication rate were China (n = 556, 43.3%), Sweden (n = 90, 7%), Canada (n = 77, 6%), and Germany (n = 75, 5.8%). Collaboration has a very strong influence on the research into CPs. Asia, North America, and Europe show strong collaborative ties in CP research, particularly between countries like China, the USA, Canada, Sweden, Switzerland, Norway, and Germany (Figure 3). This cooperation may stem from the inclusion of SCCPs in international agreements, such as the United Nations Economic Commission for Europe (UNECE) Long-Range Transboundary Air Pollution (LRTAP) Convention, Aarhus Protocol on POPs (adopted on 18 December 2009), which has continued to track these contaminants and continues to monitor and regulate these contaminants globally (Lipnick and Muir, 2000). Additionally, Environment and Climate Change Canada and the US EPA released a joint risk management strategy in 2021 for SCCP control in the Great Lakes, enforcing federal policies to limit contaminant release (UNEP. SC-8/11). South Africa has engaged in CP-related research collaborations with European countries such as Sweden, Germany, Denmark, Norway, and France (Figure 3), likely due to its recognition as a CP producer under the Stockholm Convention (Guida et al., 2020). Support from various institutions has contributed to the growth of research in developed countries. On 26 July 2006, the European Community and its Member States proposed SCCPs for inclusion in Annexes A, B, or C of the Stockholm Convention as Parties to the Convention (UNEP/POPS/POPRC.2/INF/6 and compiled in UNEP/POPS/POPRC.2/14). Environment Canada and the UK’s Department for Environment, Food & Rural Affairs (DEFRA) also prepared risk assessment documents. International collaboration has expanded in Europe, Asia, and North America since this nomination, which has contributed to boosting awareness of CPs as hazardous compounds across the globe. These collaborations involved significant institutions that focused on various aspects of SCCPs, including their classification as possible carcinogens (Group 2B) by the International Agency for Research on Cancer (IARC), and their designation as contaminants of very high concern under the EU’s Registration, Evaluation, Authorisation, and Restriction of Chemicals (REACH) guideline due to their impact on the liver, thyroid, and kidneys. The UNECE Aarhus (POPs) Protocol to the Convention on LRTAP also recognized SCCPs as POPs, and the Oslo-Paris (OSPAR) Commission regulated their main uses and sources through Decision 95/1 for the safeguarding of Marine Environment of the North-East Atlantic. In November 2007, SCCPs were further included in the Baltic Sea Action Plan by the Baltic Marine Environment Protection Commission (HELCOM), aiming to restrict the use of hazardous substances, including SCCPs, in the Baltic Sea region by 2008 (UNEP, 2022; Glüge et al., 2018; Krätschmer et al., 2023; Schächtele et al., 2023). The global campaign to regulate SCCPs extended to the UK, EU, Switzerland, Australia, and Canada, with attention also being drawn to MCCPs due to their persistent, bioaccumulative, and toxic properties (Yuan et al., 2023); although their bioaccumulative behaviours are still unclear (Nevondo and Okonkwo, 2021). Given these collaborations, it is unsurprising that research from these countries has contributed to a substantial number of publications. Most of the research on human exposure involved collaborations among eighty-two United Nations member states, with fifty nations participating in multiple studies from 2000 to 2019. These efforts focused on assessing the effectiveness of the Stockholm Convention in reducing or eliminating emissions of chlorinated POPs. These exposure studies on human milk were carried out at the State Institute for Chemical and Veterinary Analysis of Food (CVUA) in Freiburg, Germany, under the direction of the World Health Organization and the United Nations Environment Programme (WHO/UNEP) (Nipen et al., 2022; Babayemi et al., 2022), and human biomonitoring and exposure collaborative research between Sweden and Norway (Wang and Gao, 2016). Other top countries in CP collaboration studies include Switzerland (n = 57, 4.4%), the USA (n = 47, 3.7%), the UK (n = 44, 3.4%), Japan (n = 38, 3%), France (n = 31, 2.4%), and Spain (n = 28, 2.2%). These countries also dominated citation metrics, except for France, which was replaced by Australia. The top six countries with the highest citation metrics were China (12,007), Canada (3,899), Sweden (3,024), Switzerland (3,017), the UK (2,482), and the USA (2,329). Most of the collaborating countries with high citation metrics were from Europe and Asia. Research from this region is well-supported with funding and equipped with the necessary facilities. In contrast, publications from Africa were scarce, with contributions from Egypt (n = 7), South Africa (n = 5), Nigeria (n = 3), Kenya (n = 1), and Liberia (n = 1), most of which were the result of international collaboration. Despite the relatively high concentrations of CPs detected in indoor dust in South Africa (Guida et al., 2020; Chen et al., 2020), human milk (Krätschmer et al., 2021), air and soil (Klaunig et al., 2003) compared to other countries, data from African countries remain limited. This scarcity of information on CPs in Africa could be due to a lack of production in most African countries, except South Africa and Egypt. Production, importation, and use of CPs are still restricted in Nigeria. However, there have been reports of unregulated and undocumented importation of large quantities of unlabelled products containing SCCPs (Zhang and Horrocks, 2003). Consequently, African and developing countries might not prioritize research on CPs since they have more pressing needs and few resources to fund the project, especially being aware that these chemicals are restricted. Moreover, Africa as a developing continent, faces challenges in collaboration and funding for research. However, projects like the Field and mechanism-based toxicity research on pesticides in Africa (ToRePs) funded by Africa-Japan Collaborative Research (AJ-CORE) in collaboration with the National Research Foundations (NRF), have advanced pesticide research in countries like South Africa, Ghana, and Zambia. These initiatives illustrate how funding and international partnerships can enhance research. Expanding such support from more institutions on CP projects could foster further collaborations between developed nations and African research bodies, empowering contributions from scientists and diverse stakeholders.

**TABLE 1:** Top 25 corresponding author countries on research conducted on the assessment of CPs across the globe from 1916 to 2024 ranked based on publication and citation indices.

Most productive countries								Total number of citations per country			
Rank	Countries	Articles	% of 1,425	Freq	SCP	MCP	MCP/P ratio	Rank	Country	Total citations	Citation average
1	China	556	43.268	0.432	484	72	0.130	1	China	12,007	21.6
2	Sweden	90	7.004	0.070	63	27	0.300	2	Canada	3,899	50.64
3	Canada	77	5.992	0.060	59	18	0.234	3	Sweden	3,024	33.6
4	Germany	75	5.837	0.058	64	11	0.147	4	Switzerland	3,017	52.93
5	Switzerland	57	4.436	0.044	42	15	0.263	5	United Kingdom	2,482	56.41
6	United States	47	3.658	0.036	38	9	0.192	6	United States	2,329	49.55
7	United Kingdom	44	3.424	0.034	37	7	0.159	7	Germany	1750	23.33
8	Japan	38	2.957	0.030	30	8	0.211	8	Spain	1,023	36.54
9	France	31	2.412	0.024	27	4	0.129	9	Australia	785	49.06
10	Spain	28	2.179	0.022	25	3	0.107	10	Japan	695	18.29
11	Norway	26	2.023	0.020	10	16	0.615	11	Norway	649	24.96
12	India	18	1.401	0.014	16	2	0.111	12	Netherlands	538	29.89
13	Netherlands	18	1.401	0.014	9	9	0.500	13	France	523	16.87
14	Australia	16	1.245	0.012	6	10	0.625	14	Denmark	345	31.36
15	Belgium	16	1.245	0.012	10	6	0.375	15	Czech Republic	334	25.69
16	Italy	15	1.167	0.012	15	0	0.000	16	Italy	304	20.27
17	Czech Republic	13	1.012	0.010	11	2	0.154	17	India	186	10.33
18	Korea	12	0.934	0.009	10	2	0.167	18	Korea	180	15
19	Denmark	11	0.856	0.009	10	1	0.091	19	Saudi Arabia	161	161
20	Russia	10	0.778	0.008	10	0	0.000	20	Belgium	146	9.12
21	Iran	9	0.700	0.007	6	3	0.333	21	Egypt	107	15.29
22	Egypt	7	0.545	0.005	7	0	0.000	22	Iran	106	11.78
23	Pakistan	6	0.467	0.005	2	4	0.667	23	Brazil	93	23.25
24	Poland	6	0.467	0.005	6	0	0.000	24	South Africa	82	16.4
25	South Africa	5	0.389	0.004	3	2	0.400	25	Singapore	72	72

SCP, single country publications; MCP, multiple country publications.

### 3.3 Publication outlets

The top 25 journals for CP research output throughout the evaluation period are shown in Table 2. Chemosphere was the leading journal, publishing 116 articles and accounting for 13.1% of the total collection. Following closely were Environmental Science & Technology and Science of the Total Environment, which ranked second and third with 114 (12.9%) and 104 (11.7%) articles, respectively. Environmental Pollution (n = 90, 10.2%) and Environment International (n = 42, 4.7%) occupied the fourth and fifth positions respectively. Between 1916 and 2024, a total of 443 journal sources featured publications on CPs. The multidisciplinary focus of these journals which publish work on chemical detection, behaviour, toxicology, and remediation across the biosphere, hydrosphere, lithosphere, and atmosphere, probably accounts for their prominence. While advocating for solutions to critical environmental issues, they investigate environmental contamination and its consequences on ecosystems and human health. These publications cover a wide range of issues, including environmental chemistry, toxicology, public health, risk assessment, and environmental technology. They are aimed towards an international readership of scientists, policymakers, and environmental professionals. The Journal of Hazardous Materials has the highest impact factor (12.2), followed by Environmental Science & Technology (10.8), Environment International (10.3), Chemosphere (8.1), and Environmental Pollution (7.6). Significantly, the primary focus of all these prominent journals is on the impact of chemicals on the environment.

**TABLE 2:** The top 25 cited publications on the assessment of CP across the globe from 1916 to 2024 ranked based on their citation frequencies.

Top manuscript per citation						Most relevant sources	
Rank	First author, initials, and year of publication	Journal Title	DOI	TC	TC/year	Sources	Articles
1	Klaunig J, 2003	Crit Rev Toxicol-A	10.1080/713,608,372	544	24.73	Chemosphere	116
3	Zhang S, 2003	Prog Polym Sci	10.1016/j.progpolymsci.2003.09.001	480	21.82	Environmental Science & Technology	114
4	Shaw S, 2010	Rev Environ Health	10.1515/REVEH.2010.25.4.261	459	30.6	Science Of the Total Environment	104
5	Braune B, 2005	Sci Total Environ	10.1016/j.scitotenv.2004.10.034	324	16.2	Environmental Pollution	90
6	Hallgren S, 2002	Toxicology	10.1016/S0300-483X(02)00,222-6	295	12.83	Journal Of Hazardous Materials	43
7	Van M L, 2016	Chemosphere	10.1016/j.chemosphere.2016.04.037	241	26.78	Environment International	42
8	Glüge J, 2016	Sci Total Environ	10.1016/j.scitotenv.2016.08.105	234	26	Journal Of Chromatography A	25
9	Tomy G, 1997	Anal Chem	10.1021/ac961244y	222	7.93	Environmental Toxicology and Chemistry	21
10	Houde M, 2008	Environ Sci Technol	10.1021/es703184s	214	12.59	Analytical Chemistry	16
11	Reth M, 2005	J Chromatogr A	10.1016/j.chroma.2005.05.061	204	10.2	Environmental Science and Pollution Research	16
12	Brown S, 2004	Environ Toxicol Chem	10.1897/03-242	204	9.71	Environmental Science and Technology	16
13	Bayen S, 2006, Environ Int	Environ Int	10.1016/j.envint.2006.05.009	199	10.74	Polymer Degradation and Stability	16
14	Jansson B, 1993	Environ Toxicol Chem-A	10.1002/etc.5620120704	196	6.12	Ecotoxicology And Environmental Safety	12
15	Tomy G, 1998	Rev Environ Contam Toxicol	10.1007/978-1-4612-1708-4_2	187	6.93	Journal Of Applied Polymer Science	11
16	Smith S, 2009	Philos Trans R Soc A-Math Phys Eng Sci	10.1098/rsta.2009.0154	179	11.19	Wear	11
17	Gluege J, 2018	Environ Sci Technol	10.1021/acs.est.7b06459	171	24.43	Analytical And Bioanalytical Chemistry	10
18	Alcock R	Chemosphere-A	10.1016/S0045-6535(98)00,444-5	168	6.46	Trac-Trends in Analytical Chemistry	10
19	Ma X, 2014	Environ Sci Technol	10.1021/es500940p	161	14.64	Ambio	9
20	Seleiman M, 2020	Resour Conserv Recycl	10.1016/j.resconrec.2019.104647	161	32.2	Archives Of Toxicology	9
21	Chen M, 2011	Environ Sci Technol	10.1021/es202891a	160	11.43	Environmental Science \\and Technology Letters	9
22	Gao Y, 2012	Environ Sci Technol	10.1021/es2041256	153	11.77	Chinese Journal Of Chromatography	8
23	Zeng L, 2011	Environ Sci Technol	10.1021/es103740v	151	10.79	Emerging Contaminants	8
24	Friden U, 2011	Environ Int	10.1016/j.envint.2011.04.002	149	10.64	Environmental Research	8
25	Alcock R, 1999	Chemosphere	10.1016/S0045-6535(98)00444-5	149	5.73	Food Chemistry	8

### 3.4 Citation records and author distribution patterns

**FIGURE 4:**
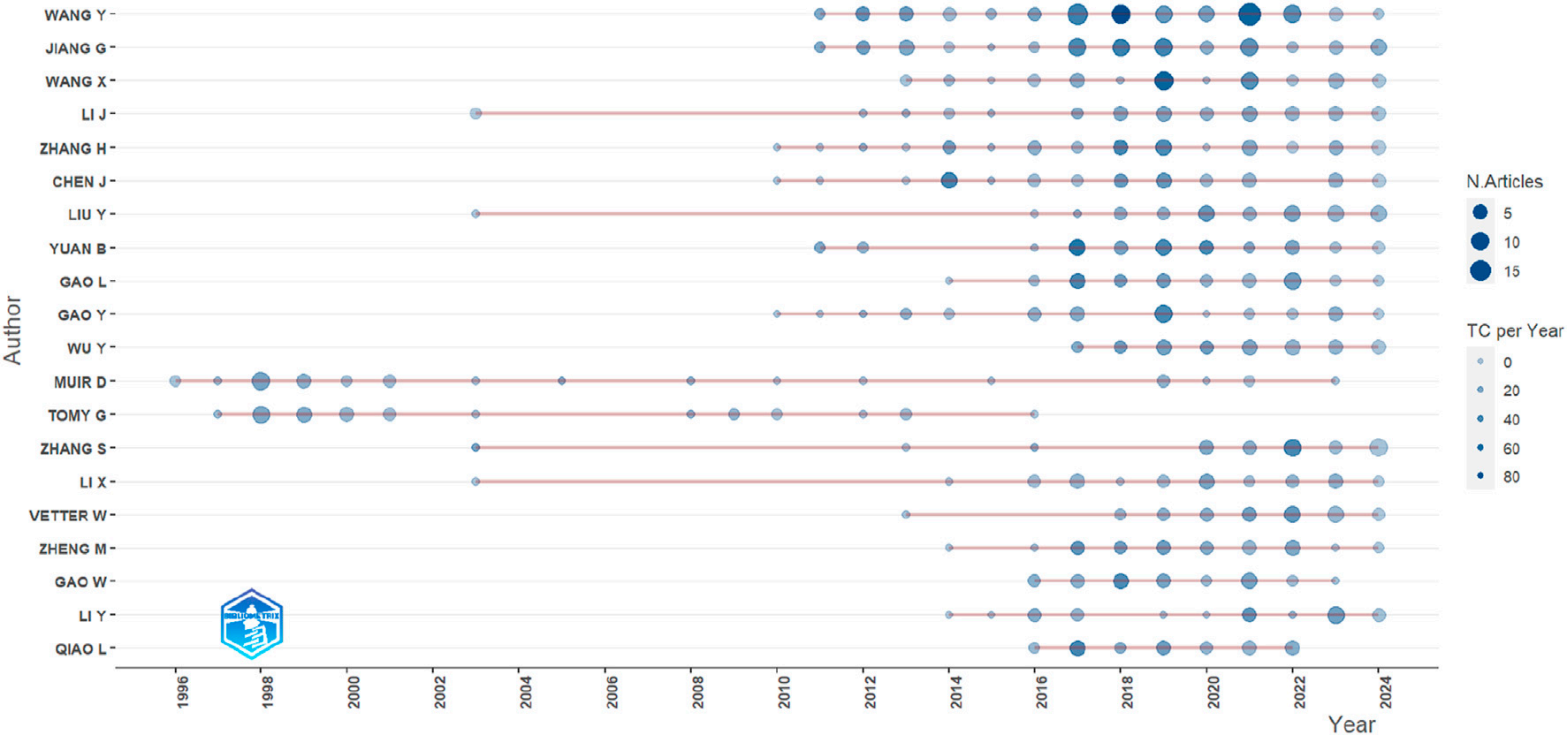
The 25 most productive scientists’ annual productivity in CP studies over time.

We analyzed citation metrics to identify the most significant articles in the collection. Highly cited literature is often recognized as groundbreaking in a research field for its original contributions and lasting impact, with frequent citations underscoring its influence. Such works also reflect trending topics and core issues within the field, highlighting their continued relevance (Shaw et al., 2010; Kwon, 2018). The top 25 most cited publications are listed in Table 2. Articles focusing on toxicology, health effects, applications, usage, toxicity, and effectiveness of CPs as flame retardants received higher citations than other topics. The article published by Klaunig et al. (2003) leads the list, with 544 citations and an average of 24.73 citations per year. This invited review article comprehensively explored the toxicology of CPs and their health effects, noting that exposure to CPs caused kidney and thyroid tumours in rats and mice. Zhang and Horrocks (2003) ranked second with 480 citations and an annual average of 21.82. Their study reviewed the application of CPs as flame retardants, highlighting how CPs lower the destabilization temperature of polypropylene. In third place is the study of Susan Shaw (Sukhareva et al., 2010), which examined the use, toxicity, and effectiveness of organohalogen fire retardants, originally thought to replace phased-out chemicals. Shaw’s research recorded 459 citations, averaging 30.6 citations per year. Her findings revealed that these fire retardants produced toxic by-products and raised environmental concerns. Of the total papers reviewed, 59% (n = 859) were published between 2015 and 2024, while 14.9% (n = 216) were published between 2003 and 2013. Figure 4 shows the productivity of the top 25 authors throughout the study period. The size of the blue bubbles represents the total number of articles, while the visibility of the bubbles reflects cumulative citations. Gaps between bubbles indicate periods of inactivity in these authors’ contributions. Among the top contributors shown in Table 3, Wang Y. (96 publications, TC = 3,243) and Jiang G. (71 publications, TC = 2,339) from the Chinese Academy of Sciences had the most papers and citations, with their work appearing between 2012 and 2024. The most recent author, Wu Y., from Sun Yat-Sen University, Guangzhou, China began publishing in 2017, amassing 37 articles by 2024. Earlier authors, such as Muir D. from Environment Canada, National Water Research Institute, Ontario, Canada (1996–2023) and Tomy G. with h-index of 29 (1997–2016) from Fisheries and Oceans Canada Freshwater Institute, Winnipeg, Canada, published 36 articles with 2,584 citations and 33 articles with 2,478 citations, respectively. Qiao L. from the University of Chinese Academy (2016–2022) had a shorter period of publication but accumulated 28 citations. Table 4 shows the top 25 authors from 1916 to 2024 ranked by authorship frequency. Only a few of these authors had single-authored articles, including Darnerud P (3) and Tomy G (1); implying that they single-handedly conceptualized and contributed to the articles. However, 97% of Tomy G’s total papers were multi-authored. All other authors contributed to multi-authored and first-authored articles. While Yuan B did not have any single-authored papers, he was the first author of 47% of his total articles. These highly referenced authors have collaborated and will likely continue to collaborate.

**TABLE 3:** Bibliometric evaluation of the top 25 most productive authors on research conducted on the assessment of CPs across the globe from 1916 to 2024 ranked based on publication, citation indices, and frequency of articles.

	Most productive authors				Author index						
	Authors	Articles	Authors	Article fractionalized	Element	h_index	g_index	m_index	TC	NP	PY_start
1	Wang Y	96	Wang Y	14.12	Bleiner D	7	13	1.00	177	17	2018
2	Jiang G	71	Jiang G	10.57	Bogdal C	17	17	1.42	1,436	17	2013
3	Wang X	46	Darnerud P	9.07	Cariou R	8	15	0.89	253	17	2016
4	Li J	45	Tomy G	8.41	Chen J	22	36	1.47	1,298	43	2010
5	Zhang H	44	Muir D	7.94	Darnerud P	13	21	0.29	683	21	1980
6	Chen J	43	Vetter W	7.6	Fisk A	16	18	0.55	1,507	18	1996
7	Liu Y	41	Yuan B	7.29	Fu J	13	22	1.00	605	22	2012
8	Yuan B	40	Coelhan M	6.92	Gao L	18	32	1.64	1,039	37	2014
9	Gao L	37	Wang X	6.39	Gao W	18	29	2.00	890	30	2016
10	Gao Y	37	Anon A	6	Gao Y	19	32	1.27	1,041	37	2010
11	Wu Y	37	Na N	6	Geng N	14	18	1.40	627	18	2015
12	Muir D	36	Li J	5.71	Heeb N	12	18	1.50	350	20	2017
13	Tomy G	33	Zhang H	5.67	Jiang G	29	47	2.07	2,339	71	2011
14	Zhang S	33	Chen J	5.65	Knobloch M	8	13	1.14	183	18	2018
15	Li X	32	Gao Y	5.59	Li	11	17	0.85	437	17	2012
16	Vetter W	32	Oehme M	5.52	Li H	16	25	1.23	632	27	2012
17	Zheng M	32	Li X	5.19	Li J	17	32	0.77	1,085	45	2003
18	Gao W	30	Liu Y	5.07	Li Q	11	19	0.69	465	19	2009
19	Li Y	29	Zitko V	5	Li X	15	21	0.68	486	32	2003
20	Qiao L	28	Zhang S	4.68	Li Y	14	23	1.27	554	29	2014
21	Li H	27	Camino G	4.67	Liu J	10	22	0.37	543	22	1998
22	Zhou Y	26	Costa L	4.67	Liu W	12	19	0.43	611	19	1997
23	Xu C	25	Gao W	4.64	Liu X	6	15	0.50	231	17	2013
24	Wang Z	23	Wu Y	4.61	Liu Y	15	25	0.68	656	41	2003
25	Zeng L	23	Sprengel J	4.45	Luo X	12	19	0.86	658	19	2011

**TABLE 4:** The top 25 cited publications on the assessment of CPs across the globe from 1916 to 2024 ranked based on their authorship pattern.

Rank	Author	Dominance factor	Tot articles	Single-authored	Multi-authored	First authored	Rank by articles	Rank by DF
1	Knobloch M	0.611	18	0	18	11	35	1
2	Darnerud P	0.556	21	3	18	10	25	2
3	Fisk A	0.556	18	0	18	10	35	2
4	Sprengel J	0.529	17	0	17	9	41	4
5	Xia D	0.500	18	0	18	9	35	5
6	Yuan B	0.475	40	0	40	19	7	6
7	Li H	0.444	27	0	27	12	18	7
8	Zhang Z	0.412	17	0	17	7	41	8
9	Zeng L	0.391	23	0	23	9	21	9
10	Liu L	0.375	16	0	16	6	46	10
11	Van Ml	0.368	19	0	19	7	31	11
12	Sun Y	0.333	21	0	21	7	25	12
13	Li Q	0.316	19	0	19	6	31	13
14	Chen C	0.313	16	0	16	5	46	14
15	Wang X	0.304	46	0	46	14	3	15
16	Tomy G	0.281	33	1	32	9	11	16
17	Wu Y	0.243	37	0	37	9	8	17
18	Geng N	0.222	18	0	18	4	35	18
19	Zhang Y	0.217	23	0	23	5	21	19
20	Gao W	0.200	30	0	30	6	15	20
21	Xu C	0.200	25	0	25	5	20	20
22	Zhou Y	0.192	26	0	26	5	19	22
23	Chen X	0.188	16	0	16	3	46	23
24	Liu X	0.176	17	0	17	3	41	24
25	Liu Y	0.171	41	0	41	7	6	25

### 3.5 Keyword analysis and thematic domain

**FIGURE 5:**
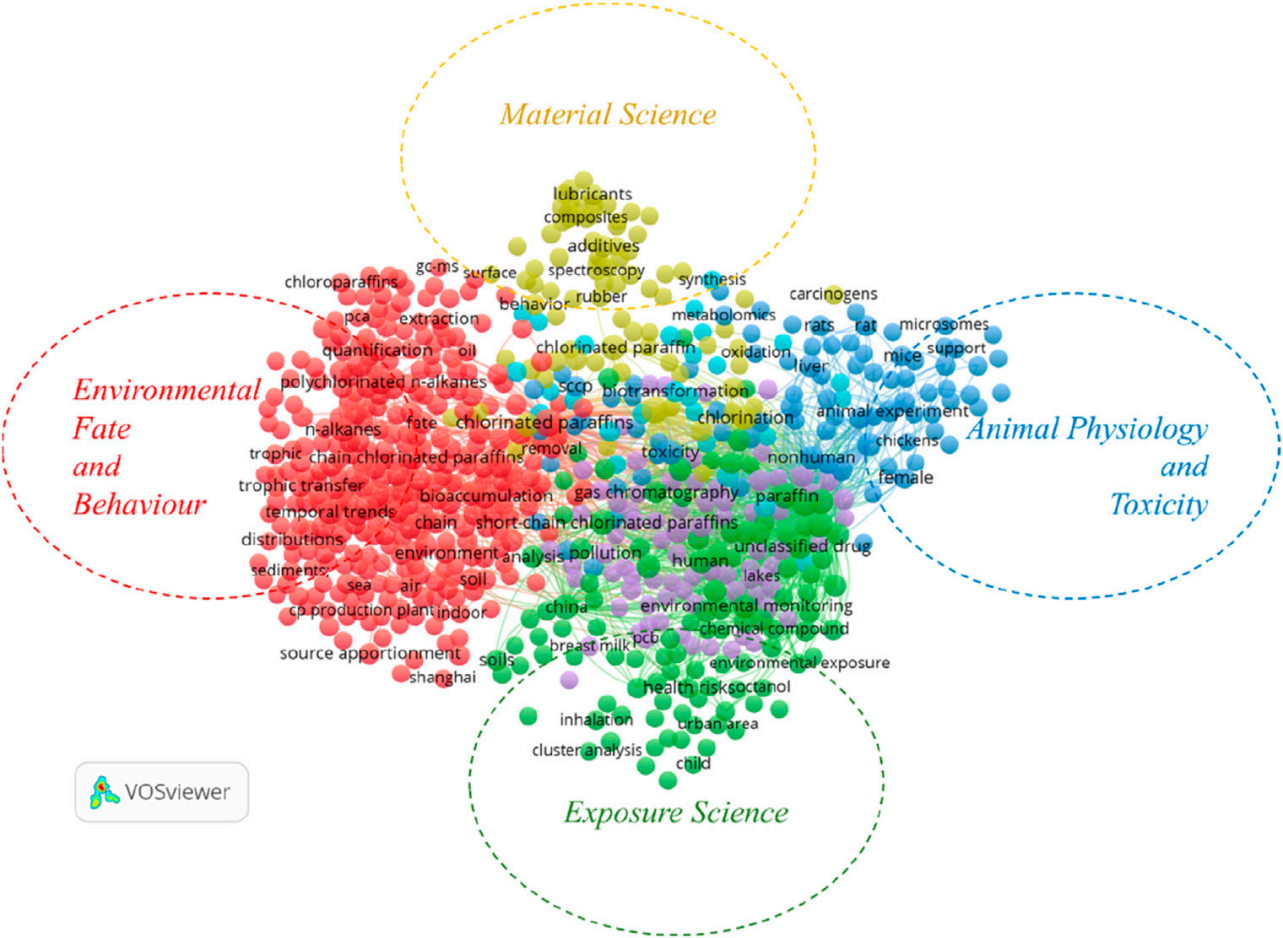
Thematic literature classification of research related to CP across the globe based on data retrieved from the WoS and Scopus databases from 1916 to 2024.

Author keywords are terms authors select to summarize and represent the content of scientific publications. They are key in data retrieval, bibliometrics, and knowledge arrangements (Li et al., 2023; Yan et al., 2023). The Top 25 most relevant keywords (DE) (2,893) and keywords-plus (ID) (5,067) based on research on CP are shown in Table 5 in a ranking of occurrence and prevalence. These search terms were applied to analyze trends in the CP literature. With 221 and 262 articles for relevant keywords (DE) and keywords-plus (ID), respectively, chlorinated paraffins and paraffin have the highest percentage of the two categories. A keyword network map (Figure 5) was generated to identify the research hotspots. Four research thematic areas of CPs were identified: Material Science, Environmental Fate and Behaviour, Animal Physiology and Toxicity, and Exposure Science.

**TABLE 5:** Top 25 most relevant keywords (DE) and keywords-plus (ID) on research conducted on the assessment of CP across the globe from 1916 to 2024 ranked based on publication.

Rank	Author keywords (DE)	Articles	% Of 1,640	Articles keywords-plus (ID)	Articles	% Of 1,640
1	Chlorinated paraffins	221	15.252	Paraffin	262	18.081
2	Short-chain chlorinated Paraffins	80	5.521	Chlorinated paraffins	176	12.146
3	SCCPs	75	5.176	China	169	11.663
4	Chlorinated paraffin	65	4.486	Paraffins	153	10.559
5	Bioaccumulation	43	2.968	Bioaccumulation	147	10.145
6	Persistent organic pollutants	39	2.692	Environmental monitoring	146	10.076
7	MCCPs	36	2.484	Article	128	8.834
8	Risk assessment	35	2.415	Polychlorinated biphenyls	122	8.420
9	Short-chain chlorinated paraffins	31	2.139	Environmental samples	119	8.213
10	Distribution	30	2.070	Polybrominated diphenyl ethers	115	7.937
11	SCCP	28	1.932	Chain chlorinated paraffins	111	7.660
12	Short-chain chlorinated paraffins (SCCPs)	28	1.932	Hydrocarbons	111	7.660
13	Polychlorinated n-alkanes	27	1.863	Polychlorinated n-alkanes	109	7.522
14	Chlorinated paraffins (CPs)	24	1.656	Chlorinated	104	7.177
15	Human exposure	24	1.656	Chlorinated hydrocarbon	103	7.108
16	Sediment	22	1.518	Sediments	96	6.625
17	Analysis	21	1.449	Exposure	94	6.487
18	Short-chain chlorinated paraffin	21	1.449	Flame retardants	92	6.349
19	Biomagnification	20	1.380	N-alkanes	89	6.142
20	Polychlorinated alkanes	20	1.380	Mass spectrometry	83	5.728
21	Soil	20	1.380	Short-chain	81	5.590
22	Mass spectrometry	17	1.173	Fish	80	5.521
23	Medium-chain chlorinated paraffins	17	1.173	Risk assessment	77	5.314
24	Fish	16	1.104	Temporal trends	76	5.245
25	MCCP	16	1.104	Toxicity	76	5.245

The mixed-colour lines that link the two phrases indicate their degree of collaboration. The use of cluster analysis which classifies items into several groups with related themes depending on their similarity was employed to describe the keyword network map (Darnerud and Bergman, 2022). With keywords like lubricants, composites, additives, and rubber, the golden cluster (cluster 1) in Figure 5 focuses on material science. CPs have been used in these materials to improve qualities like flexibility, fire resistance, and chemical resistance, which makes them important for industrial applications (He et al., 2023; Yang et al., 2021; NTP, 1986; Chen et al., 2024). Cheap commercial production of chlorinated paraffins and their huge industrial applications in various fields started in the 1920s and 1932 without restrictions on the use of MCCPs and LCCPs (Vetter et al., 2022; Guida et al., 2020), with an estimated 33 million metric tons of CPs generated and consumed worldwide by 2020 (Chen C. et al., 2022), and predicted production increase in the foreseeable future (Yuan S. et al., 2021). The enormous applications would have encouraged researchers to further their investigation in material science.

The blue cluster (cluster 2) is depicted as Animal Physiology and Toxicity with keywords such as microsome, mice, support, animal experiment, female, chicken, non-human, rat, and liver. However, little research exists in this niche compared to other POPs (Chen et al., 2023), and there is currently no metabolic scheme provided, and the metabolic transformations of CPs are not well-explored (Fernandes et al., 2022b). Over the past few years, studies on the toxicity of SCCPs to zebrafish and their cells have employed metabolomics based on high-resolution mass spectrometry, which provides a quantitative research methodology. However, many of the physiological mechanisms in zebrafish differ significantly from those in mammals, posing challenges for direct comparisons (107). The green cluster (cluster 3), focused on Exposure Science with keywords such as child, inhalation, urban area, health risks, environmental exposure, soil, China, octanol, and cluster analysis. However, most studies on health and exposure were conducted *in vitro* as epidemiological research is scarce; therefore, future studies on human exposure and adverse effects of CPs should use more exposure methodologies (Huang et al., 2023; Xu et al., 2021). Environmental Fate and Behaviour are the red clusters (cluster 4), with the keywords that include source apportionment, CP production, Shanghai, plant, indoor, sediment, distribution, temporal trends, tropical chain, chlorinated paraffin, GC-MS, extraction, and quantification. This focuses on publications that promote the study of harmful substances and their interactions with the environment. A significant toxicology study on CPs, conducted by the US National Toxicology Program, NTP (1986), demonstrated carcinogenic effects on the kidneys, liver, and thyroid in rodents. CP toxicology research from 1900 to 2023 reported by Chen et al. (2023) reveals that only 13% of studies focused on the analytical chemistry of CPs, while 7% addressed toxicology. Notably, just 26% of the toxicological research was published between 2014 and 2023, suggesting a significant gap in both toxicology and analytical chemistry data for CPs. The study further suggests that computational toxicology could serve as a more cost-effective alternative to traditional *in vivo* experiments. Most of these studies were conducted in China, considering their position in Table 1. This could probably be due to their involvement in mass production (Pan et al., 2021). Recently, there has been a growing interest in very short CPs (vSCCPs, C < 10), which are environmental byproducts produced during the manufacture of CPs (Chen et al., 2024).

## 4 Future perspectives and recommendations

Since CPs were designated as POPs under the Stockholm Convention due to their harmful environmental and health effects, research interest has surged globally. This scientometric analysis indicates a steady annual increase in CP-related publications, with an average growth rate of 4.3%, reflecting a significant rise in article output. It went from just one publication in 1916 to 106 in 2023, suggesting continued growth in future research. This growth would be enhanced by the vast collaborations and funding that these studies have attracted.

Compared to legacy POPs like polychlorinated biphenyls (PCBs), dioxins, and DDT, global CP production is considerably higher, with an estimated 32.5 million tons produced in 2020, and CPs continue to be manufactured and used in some developing countries (Lyu and Zhang, 2023; Mu et al., 2023). This increasing production and usage leads to an increasing risk of environmental pollution and adverse effects on human health. Therefore, regulatory bodies should enhance monitoring and implement stricter controls on CP emissions. In this regard, special attention is needed for developing countries that lack funding, analytical capacity, and waste management techniques, as these gaps may worsen pollution (Guida et al., 2020). Also, the regulation of CPs should be expedited by international cooperation and partnerships in tackling the global health and environmental concerns relating to these chemicals through information transfer (Krätschmer et al., 2023). Additionally, the Stockholm Convention’s restrictions on recycling SCCP-containing products pose significant challenges to waste management, particularly for developing countries with limited disposal and analytical capacities. This is because SCCP recycling may lead to the release of other hazardous substances such as PCBs and polychlorinated naphthalenes (Yuan S. et al., 2021; Guida et al., 2020).

CPs present analytical challenges, including poor detection sensitivity, especially for compounds with <4 chlorine atoms (Fernandes et al., 2022a; Yuan B. et al., 2021b). This challenge can also be due to unsuitable reagents for CP analysis. To improve accuracy, a range of reagents should be employed to cover various carbon chain lengths and chlorine levels (Huang et al., 2023). Additionally, organizing laboratory exchange visits and training sessions on advanced analytical techniques and instrumentation is essential for enhancing CP research proficiency among researchers.

Although MCCP and LCCP concentrations in human and environmental samples are now rapidly exceeding those of SCCPs (Chen et al., 2024; Huang et al., 2023), their levels and environmental fate remain largely unknown, especially as their production increases to replace SCCP products (South et al., 2022). Expanding global environmental data on LCCPs, particularly beyond China, is essential to fill existing research gaps and gain a clearer understanding of LCCP transformation, ecological transfer, and impact, especially in the marine environment (Chen et al., 2024; Lyu and Zhang, 2023; Yuan et al., 2022b). Future studies should prioritize LCCP bioaccumulation and toxicity across various food webs, focusing on aquatic species vulnerable to CPs, which also serve as effective models for toxicological studies (Huang et al., 2023; Chen et al., 2023). Enhanced research into CP biodegradation pathways in natural environments is necessary, and enzyme-based biosynthesis presents a promising approach for studying the toxicity of CP metabolites (Chen et al., 2024).

## 5 Conclusion

This study examined CP-related publications from 1916 to 2024, using data from the Web of Science and Scopus to assess research trends and the status of the field. Although global CP use was restricted by the Stockholm Convention in 2017, publications in this field have increased significantly, with North America, Asia, and Europe leading in citation impact and research collaboration. However, studies from developing countries CPs remain limited, highlighting the need for additional regional programs, funding, and partnerships. An average annual growth rate of 4.3% indicates expected growth in CP-related publications. VOSviewer analysis showed that SCCP research dominates CP literature. However, there is a pressing need for more studies on MCCPs and LCCPs, especially from developing nations, to enhance the global response under the Stockholm Convention.

## References

[B1] Adetunji T. L. Olawale F. Olisah C. Adetunji A. E. Aremu A. O. C. (2022). Capsaicin: a two-decade systematic review of global research output and recent advances against human cancer. Front. Oncol. 12, 908487. Systematic Review. 10.3389/fonc.2022.908487 35912207 PMC9326111

[B2] Alipour Parvizian B. Fernando S. Crimmins B. S. Hopke P. K. Holsen T. M. (2024). Trends of short- and medium-chain chlorinated paraffin concentrations in top predator fish tissues from the Great Lakes. ACS ES&T Water 4 (8), 3433–3442. 10.1021/acsestwater.4c00264

[B4] Aravind Kumar J. Krithiga T. Sathish S. Renita A. A. Prabu D. Lokesh S. (2022). Persistent organic pollutants in water resources: fate, occurrence, characterization and risk analysis. Sci. Total Environ. 831, 154808. 10.1016/j.scitotenv.2022.154808 35341870

[B5] Aria M. Cuccurullo C. bibliometrix (2017). Bibliometrix: an R-tool for comprehensive science mapping analysis. J. Inf. 11 (4), 959–975. 10.1016/j.joi.2017.08.007

[B6] Arko W. E. Zhao S. Ma J. Tian L. Asante K. A. Amoah D. K. (2024). Impact of anthropogenic activities on atmospheric chlorinated paraffins in Ghana using polyurethane foam disk - passive air sampler. Sci. Total Environ. 954, 176252. 10.1016/j.scitotenv.2024.176252 39278497

[B7] Babayemi J. O. Nnorom I. C. Weber R. (2022). Initial assessment of imports of chlorinated paraffins into Nigeria and the need of improvement of the Stockholm and Rotterdam Conventions. Emerg. Contam. 8, 360–370. 10.1016/j.emcon.2022.07.004

[B8] Beloki Ezker I. Yuan B. Bohlin-Nizzetto P. Borgen A. R. Wang T. (2024). Polychlorinated alkanes in indoor environment: a review of levels, sources, exposure, and health implications for chlorinated paraffin mixtures. Chemosphere 365, 143326. 10.1016/j.chemosphere.2024.143326 39306115

[B9] Chain E. P. o. C. i. t. F. Schrenk D. Bignami M. Bodin L. Chipman J. K. del Mazo J. (2020). Risk assessment of chlorinated paraffins in feed and food. EFSA J. 18 (3), e05991. 10.2903/j.efsa.2020.5991 32874241 PMC7447893

[B10] Chen C. (2014). The citespace manual. Coll. Comput. Inf. 1 (1), 1–84.

[B11] Chen C. Chen A. Zhan F. Wania F. Zhang S. Li L. (2022a). Global historical production, use, in-use stocks, and emissions of short-medium-and long-chain chlorinated paraffins. Environ. Sci. and Technol. 56 (12), 7895–7904. 10.1021/acs.est.2c00264 35536664

[B13] Chen L. Mai B. Luo X. (2022b). Bioaccumulation and biotransformation of chlorinated paraffins. Toxics 10 (12), 778. 10.3390/toxics10120778 36548610 PMC9783579

[B14] Chen L. Wei Q. Li J. Liao D. Feng D. (2020). A scientometric visualization analysis for global toxicology and pharmacology research of natural products from 1962 to 2018. Phytomedicine 68, 153190. 10.1016/j.phymed.2020.153190 32109739

[B15] Chen S. Gong Y. Luo Y. Cao R. Yang J. Cheng L. (2023). Toxic effects and toxicological mechanisms of chlorinated paraffins: a review for insight into species sensitivity and toxicity difference. Environ. Int. 178, 108020. 10.1016/j.envint.2023.108020 37354881

[B16] Chen W. Liu J. Hou X. Jiang G. (2024). A review on biological occurrence, bioaccumulation, transmission and metabolism of chlorinated paraffins. Crit. Rev. Environ. Sci. Technol. 54 (5), 424–443. 10.1080/10643389.2023.2246615

[B17] Cui L. Gao L. Zheng M. Li J. Zhang L. Wu Y. (2020). Short- and medium-chain chlorinated paraffins in foods from the sixth Chinese total diet study: occurrences and estimates of dietary intakes in South China. J. Agric. Food Chem. 68 (34), 9043–9051. 10.1021/acs.jafc.0c03491 32786846

[B18] Darnerud P. O. Bergman Å. (2022). Critical review on disposition of chlorinated paraffins in animals and humans. Environ. Int. 163, 107195. 10.1016/j.envint.2022.107195 35447436

[B19] Ding L. Luo N. Liu Y. Fang X. Zhang S. Li S. (2020). Short and medium-chain chlorinated paraffins in serum from residents aged from 50 to 84 in Jinan, China: occurrence, composition and association with hematologic parameters. Sci. Total Environ. 728, 137998. 10.1016/j.scitotenv.2020.137998 32361102

[B20] Ding L. Zhang S. Zhu Y. Zhao N. Yan W. Li Y. (2021). Overlooked long-chain chlorinated paraffin (LCCP) contamination in foodstuff from China. Sci. Total Environ. 801, 149775. 10.1016/j.scitotenv.2021.149775 34467914

[B21] Dong S. Zhang S. Li X. Wei S. Li T. Zou Y. (2020). Occurrence of short- and medium-chain chlorinated paraffins in raw dairy cow milk from five Chinese provinces. Environ. Int. 136, 105466. 10.1016/j.envint.2020.105466 31935560

[B22] Feo M. L. Eljarrat E. Barceló D. Barceló D. (2009). Occurrence, fate and analysis of polychlorinated n-alkanes in the environment. TrAC Trends Anal. Chem. 28 (6), 778–791. 10.1016/j.trac.2009.04.009

[B23] Fernandes A. R. Falandysz J. Yuan B. (2022b). Widening knowledge horizons on legacy POPs: chlorinated paraffins and polychlorinated naphthalenes. Chemosphere 289, 133131. 10.1016/j.chemosphere.2021.133131 34863731

[B24] Fernandes A. R. Vetter W. Dirks C. van Mourik L. Cariou R. Sprengel J. (2022a). Determination of chlorinated paraffins (CPs): analytical conundrums and the pressing need for reliable and relevant standards. Chemosphere 286, 131878. 10.1016/j.chemosphere.2021.131878 34416588

[B25] Fiedler H. (2010). “Short-chain chlorinated paraffins: production, use and international regulations,” in Chlorinated paraffins. Editor Boer J. (Springer Berlin Heidelberg), 1–40.

[B26] Gao W. Lin Y. Liang Y. Wang Y. Jiang L. Wang Y. (2021). Percutaneous penetration and dermal exposure risk assessment of chlorinated paraffins. J. Hazard. Mater. 416, 126178. 10.1016/j.jhazmat.2021.126178 34492952

[B27] Girones L. Guida Y. Oliva A. L. Machado Torres J. P. Marcovecchio J. E. Vetter W. (2023). Short- and medium-chain chlorinated paraffins in fish from an anthropized south-western Atlantic estuary, Bahía Blanca, Argentina. Chemosphere 328, 138575. 10.1016/j.chemosphere.2023.138575 37011823

[B28] Glüge J. Schinkel L. Hungerbühler K. Cariou R. Bogdal C. (2018). Environmental risks of medium-chain chlorinated paraffins (MCCPs): a review. Environ. Sci. and Technol. 52 (12), 6743–6760. 10.1021/acs.est.7b06459 29791144

[B29] Glüge J. Wang Z. Bogdal C. Scheringer M. Hungerbühler K. (2016). Global production, use, and emission volumes of short-chain chlorinated paraffins – a minimum scenario. Sci. Total Environ. 573, 1132–1146. 10.1016/j.scitotenv.2016.08.105 27701008

[B30] Gong P. Xu H. Wang C. Chen Y. Guo L. Wang X. (2021). Persistent organic pollutant cycling in forests. Nat. Rev. Earth and Environ. 2 (3), 182–197. 10.1038/s43017-020-00137-5

[B31] Guan K.-L. Luo X.-J. Lu Q.-H. Huang C.-C. Qi X.-M. Zeng Y.-H. (2023). Occurrence, spatial distribution, and risk assessment of short- and medium-chain chlorinated paraffins in sediment from black-odorous rivers across China. Chemosphere 313, 137454. 10.1016/j.chemosphere.2022.137454 36470357

[B32] Guida Y. Capella R. Weber R. (2020). Chlorinated paraffins in the technosphere: a review of available information and data gaps demonstrating the need to support the Stockholm Convention implementation. Emerg. Contam. 6, 143–154. 10.1016/j.emcon.2020.03.003

[B33] Han X. Chen H. Deng M. Du B. Zeng L. (2021). Chlorinated paraffins in infant foods from the Chinese market and estimated dietary intake by infants. J. Hazard. Mater. 411, 125073. 10.1016/j.jhazmat.2021.125073 33454569

[B34] Han X. Chen H. Zhou W. Liang B. Pang S. Du B. (2024). Occurrence, distribution and annual emissions of chlorinated paraffins in hazardous byproducts from municipal solid waste incineration plants in South China. Sci. Total Environ. 925, 171764. 10.1016/j.scitotenv.2024.171764 38494033

[B35] He W. Sun P. Zhao Y. Pu Q. Yang H. Hao N. (2023). Source toxicity characteristics of short- and medium-chain chlorinated paraffin in multi-environmental media: product source toxicity, molecular source toxicity and food chain migration control through silica methods. Sci. Total Environ. 876, 162861. 10.1016/j.scitotenv.2023.162861 36931521

[B36] Hu H. Jin H. Li T. Guo Y. Wu P. Xu K. (2022). Spatial distribution, partitioning, and ecological risk of short chain chlorinated paraffins in seawater and sediment from East China Sea. Sci. Total Environ. 811, 151932. 10.1016/j.scitotenv.2021.151932 34838909

[B37] Huang J.-W. Bai Y.-Y. Zeeshan M. Liu R.-Q. Dong G.-H. (2023). Effects of exposure to chlorinated paraffins on human health: a scoping review. Sci. Total Environ. 886, 163953. 10.1016/j.scitotenv.2023.163953 37164081

[B38] Jain M. Khan S. A. Sharma K. Jadhao P. R. Pant K. K. Ziora Z. M. (2022). Current perspective of innovative strategies for bioremediation of organic pollutants from wastewater. Bioresour. Technol. 344, 126305. 10.1016/j.biortech.2021.126305 34752892

[B39] Jones K. C. (2021). Persistent organic pollutants (POPs) and related chemicals in the global environment: some personal reflections. Environ. Sci. and Technol. 55 (14), 9400–9412. 10.1021/acs.est.0c08093 33615776

[B40] Joshi A. (2016). Comparison between Scopus and ISI web of science. J. Glob. Values 7 (1), 1–11.

[B41] Klaunig J. E. Babich M. A. Baetcke K. P. Cook J. C. Corton J. C. David R. M. (2003). PPARalpha agonist-induced rodent tumors: modes of action and human relevance. Crit. Rev. Toxicol. 33 (6), 655–780. 10.1080/713608372 14727734

[B42] Krätschmer K. Malisch R. Vetter W. Chlorinated paraffin levels in relation to other persistent organic pollutants found in pooled human milk samples from primiparous mothers in 53 countries. Environ. Health Perspect. 2021, 129 (8), 087004. 10.1289/EHP7696 34405702 PMC8371996

[B43] Krätschmer K. Schächtele A. (2019). Interlaboratory studies on chlorinated paraffins: evaluation of different methods for food matrices. Chemosphere 234, 252–259. 10.1016/j.chemosphere.2019.06.022 31220658

[B44] Krätschmer K. Vetter W. Kalina J. Malisch R. (2023). “WHO- and UNEP-coordinated human milk studies 2000–2019: findings of chlorinated paraffins,” in Persistent organic pollutants in human milk. Editors Malisch R. Fürst P. Šebková K. (Springer International Publishing), 343–382.

[B45] Kwon S. (2018). Characteristics of interdisciplinary research in author keywords appearing in Korean journals. Malays. J. Libr. Inf. Sci. 23 (2), 77–93.

[B46] Lee C.-C. Wu Y.-Y. Chen C. S. Tien C.-J. (2022). Spatiotemporal distribution and risk assessment of short-chain chlorinated paraffins in 30 major rivers in Taiwan. Sci. Total Environ. 806, 150969. 10.1016/j.scitotenv.2021.150969 34656600

[B47] Lee S. Choo G. Ekpe O. D. Kim J. Oh J.-E. (2020). Short-chain chlorinated paraffins in various foods from Republic of Korea: levels, congener patterns, and human dietary exposure. Environ. Pollut. 263, 114520. 10.1016/j.envpol.2020.114520 32283402

[B48] Li H. Gao S. Yang M. Zhang F. Cao L. Xie H. (2020). Dietary exposure and risk assessment of short-chain chlorinated paraffins in supermarket fresh products in Jinan, China. Chemosphere 244, 125393. 10.1016/j.chemosphere.2019.125393 31790997

[B49] Li Q. Cheng L. Jin X. Liu L. Shangguan J. Chang S. (2023). Chlorinated paraffins in multimedia during residential interior finishing: occurrences, behavior, and health risk. Environ. Int. 178, 108072. 10.1016/j.envint.2023.108072 37406371

[B50] Liao H. Li X. Zhou Y. Wu Y. Cao Y. Yang J. (2023). Biomonitoring, exposure routes and risk assessment of chlorinated paraffins in humans: a mini-review. Environ. Sci. Process. and Impacts 25 (10), 1588–1603. 10.1039/d3em00235g 37655634

[B51] Lipnick R. L. Muir D. C. G. (2000). History of persistent, bioaccumulative, and toxic chemicals. ACS Symp. Ser. 772, 1–12. 10.1021/bk-2001-0772.ch001 American Chemical Society

[B52] Liu Y. Aamir M. Li M. Liu K. Hu Y. Liu N. (2020). Prenatal and postnatal exposure risk assessment of chlorinated paraffins in mothers and neonates: occurrence, congener profile, and transfer behavior. J. Hazard. Mater. 395, 122660. 10.1016/j.jhazmat.2020.122660 32344298

[B53] Lu W. Liu Z. Huang Y. Bu Y. Li X. Cheng Q. (2020). How do authors select keywords? A preliminary study of author keyword selection behavior. J. Inf. 14 (4), 101066. 10.1016/j.joi.2020.101066

[B55] Lyu L. Zhang S. (2023). Chlorinated paraffin pollution in the marine environment. Environ. Sci. and Technol. 57 (32), 11687–11703. 10.1021/acs.est.3c02316 37503949

[B56] McGrath T. J. Fujii Y. Jeong Y. Bombeke J. Covaci A. Poma G. (2022). Levels of short- and medium-chain chlorinated paraffins in edible insects and implications for human exposure. Environ. Sci. and Technol. 56 (18), 13212–13221. 10.1021/acs.est.2c03255 35969810

[B57] McGrath T. J. Poma G. Matsukami H. Malarvannan G. Kajiwara N. Covaci A. (2021). Short- and medium-chain chlorinated paraffins in polyvinylchloride and rubber consumer products and toys purchased on the Belgian market. Int. J. Environ. Res. Public Health 18 (3), 1069. 10.3390/ijerph18031069 33530429 PMC7908593

[B59] Mézière M. Marchand P. Larvor F. Baéza E. Le Bizec B. Dervilly G. (2021). Accumulation of short-medium-and long-chain chlorinated paraffins in tissues of laying hens after dietary exposure. Food Chem. 351, 129289. 10.1016/j.foodchem.2021.129289 33621922

[B60] Mu Y.-W. Cheng D. Zhang C.-L. Zhao X.-L. Zeng T. (2023). The potential health risks of short-chain chlorinated paraffin: a mini-review from a toxicological perspective. Sci. Total Environ. 872, 162187. 10.1016/j.scitotenv.2023.162187 36781137

[B61] Nevondo V. Okonkwo O. J. (2021). Status of short-chain chlorinated paraffins in matrices and research gap priorities in Africa: a review. Environ. Sci. Pollut. Res. 28 (38), 52844–52861. 10.1007/s11356-021-15924-w PMC8476396 34478051

[B62] Nipen M. Vogt R. D. Bohlin-Nizzetto P. Borgå K. Mwakalapa E. B. Borgen A. R. (2022). Spatial trends of chlorinated paraffins and dechloranes in air and soil in a tropical urban, suburban, and rural environment. Environ. Pollut. 292, 118298. 10.1016/j.envpol.2021.118298 34626702

[B63] Niu S. Chen X. Chen R. Zou Y. Zhang Z. Li L. (2023). Understanding inter-individual variability in short-chain chlorinated paraffin concentrations in human blood. J. Hazard. Mater. 443, 130235. 10.1016/j.jhazmat.2022.130235 36368064

[B64] NTP (1986). NTP toxicology and carcinogenesis studies of chlorinated paraffins (C12, 60% chlorine) (CAS No. 108171-26-2*) in F344/N rats and B6C3F1 mice (gavage studies). Natl. Toxicol. Program Tech. Rep. Ser. 308, 1–206. From NLM. 12748721

[B65] Ohoro C. R. Adeniji A. O. Okoh A. I. Okoh O. O. (2022). Spatial monitoring and health risk assessment of polybrominated diphenyl ethers in environmental matrices from an industrialized impacted canal in South Africa. Environ. Geochem. Health 44 (10), 3409–3424. 10.1007/s10653-021-01114-7 34609624

[B66] Olisah C. Malloum A. Adegoke K. A. Ighalo J. O. Conradie J. Ohoro C. R. (2024). Scientometric trends and knowledge maps of global polychlorinated naphthalenes research over the past four decades. Environ. Pollut. 357, 124407. 10.1016/j.envpol.2024.124407 38908679

[B67] Pan X. Zhen X. Tian C. Tang J. (2021). Distributions, transports and fates of short- and medium-chain chlorinated paraffins in a typical river-estuary system. Sci. Total Environ. 751, 141769. 10.1016/j.scitotenv.2020.141769 32882559

[B68] Perkons I. Abdulajeva E. Bartkiene E. Zacs D. (2022). Short- and medium-chain chlorinated paraffins in commercial complementary baby food produced in different European countries: occurrence, congener group profiles, portion-based dietary intake, and risk assessment. Sci. Total Environ. 814, 152733. 10.1016/j.scitotenv.2021.152733 34973313

[B69] Reth M. Oehme M. (2004). Limitations of low resolution mass spectrometry in the electron capture negative ionization mode for the analysis of short- and medium-chain chlorinated paraffins. Anal. Bioanal. Chem. 378 (7), 1741–1747. 10.1007/s00216-004-2546-9 14997265

[B70] Schächtele A. Hardebusch B. Krätschmer K. Tschiggfrei K. Zwickel T. Malisch R. (2023). “Analysis and quality control of WHO- and UNEP-coordinated human milk studies 2000–2019: polybrominated diphenyl ethers, hexabromocyclododecanes, chlorinated paraffins and polychlorinated naphthalenes,” in Persistent organic pollutants in human milk. Editors Malisch R. Fürst P. Šebková K. (Springer International Publishing), 145–183.

[B71] Shaw S. Blum A. Weber R. Kannan K. Rich D. Lucas D. Halogenated flame retardants: do the fire safety benefits justify the risks? Rev. Environ. Health 2010, 25 (4), 261–305. 10.1515/REVEH.2010.25.4.261 (acccessed 2024-10-16). 21268442

[B72] Siamaki S. Geraei E. Zare- Farashbandi F. (2014). A study on scientific collaboration and co-authorship patterns in library and information science studies in Iran between 2005 and 2009. J. Educ. Health Promot. 3 (1), 99. 10.4103/2277-9531.139681 25250365 PMC4165092

[B73] South L. Saini A. Harner T. Niu S. Parnis J. M. Mastin J. (2022). Medium- and long-chain chlorinated paraffins in air: a review of levels, physicochemical properties, and analytical considerations. Sci. Total Environ. 843, 157094. 10.1016/j.scitotenv.2022.157094 35779735

[B74] Sukhareva K. Mikhailov I. Mamin E. Popov A. (2010). “Effect of blend ratio on rheological and mechanical properties of butadiene rubber,” in Ethylene propylene diene rubber and chlorinated paraffins blends.

[B75] Tahir A. Abbasi N. A. He C. Ahmad S. R. (2024a). Exposure and human health risk assessment of chlorinated paraffins in indoor and outdoor dust from a metropolitan city, Lahore, Pakistan. Chemosphere 347, 140687. 10.1016/j.chemosphere.2023.140687 37952823

[B76] Tahir A. Abbasi N. A. He C. Ahmad S. R. Baqar M. Qadir A. (2024b). Spatial distribution and ecological risk assessment of short and medium chain chlorinated paraffins in water and sediments of river Ravi, Pakistan. Sci. Total Environ. 926, 171964. 10.1016/j.scitotenv.2024.171964 38537810

[B77] Tomasko J. Stupak M. Hajslova J. Pulkrabova J. (2021). Application of the GC-HRMS based method for monitoring of short- and medium-chain chlorinated paraffins in vegetable oils and fish. Food Chem. 355, 129640. 10.1016/j.foodchem.2021.129640 33799253

[B78] Tomy G. T. (2010). “Analysis of chlorinated paraffins in environmental matrices: the ultimate challenge for the analytical chemist,” in Chlorinated paraffins. Editor Boer J. (Springer Berlin Heidelberg), 83–106.

[B79] UK Government Department of Environment Food and Rural Affairs (2024). Persistent organic pollutants (POPs): policy information. Available at: https://www.gov.uk/government/publications/persistent-organic-pollutants-pops-policy-information/persistent-organic-pollutants-pops-policy-information#medium-chain-chlorinated-paraffins-mccps (Accessed December 18, 2024).

[B80] UNEP (2015). in Report of the persistent organic pollutants review committee on the work of its eleventh meeting.UNEP/POPS/POPRC.11/10/Add.2

[B81] UNEP. Stockholm convention on persistent organic pollutants (POPs). 2017.

[B82] UNEP (2022). Stockholm convention on persistent organic pollutants. UNEP/POPS/POPRC.17/6 UNEP Geneva.

[B84] van Mourik L. M. Gaus C. Leonards P. E. G. de Boer J. (2016). Chlorinated paraffins in the environment: a review on their production, fate, levels and trends between 2010 and 2015. Chemosphere 155, 415–428. 10.1016/j.chemosphere.2016.04.037 27135701

[B85] Vetter W. Sprengel J. Krätschmer K. (2022). Chlorinated paraffins – a historical consideration including remarks on their complexity. Chemosphere 287, 132032. 10.1016/j.chemosphere.2021.132032 34523451

[B86] Vorkamp K. Balmer J. Hung H. Letcher R. J. Rigét F. F. (2019). A review of chlorinated paraffin contamination in Arctic ecosystems. Emerg. Contam. 5, 219–231. 10.1016/j.emcon.2019.06.001

[B87] Wang H. Chang H. Zhang C. Feng C. Wu F. (2021). Occurrence of chlorinated paraffins in a wetland ecosystem: removal and distribution in plants and sediments. Environ. Sci. and Technol. 55 (2), 994–1003. 10.1021/acs.est.0c05694 33415977

[B88] Wang K. Gao L. Zhu S. Cui L. Qiao L. Xu C. (2020a). Spatial distributions and homolog profiles of chlorinated nonane paraffins, and short and medium chain chlorinated paraffins in soils from Yunnan, China. Chemosphere 247, 125855. 10.1016/j.chemosphere.2020.125855 31935577

[B89] Wang R. Lin Y. Le S. Lu D. Gao L. Feng C. (2024). Short- and medium-chain chlorinated paraffins in breast milk in Shanghai, China: occurrence, characteristics, and risk assessment. Environ. Pollut. 347, 123690. 10.1016/j.envpol.2024.123690 38452837

[B90] Wang W. Wang J. Nie H. Fan R. Huang Y. (2020b). Occurrence, trophic magnification and potential risk of short-chain chlorinated paraffins in coral reef fish from the Nansha Islands, South China Sea. Sci. Total Environ. 739, 140084. 10.1016/j.scitotenv.2020.140084 32554110

[B91] Wang X. Gao J. (2016). Analysis of corpus-based translation practice on CiteSpace-supported foreign language teaching. Assoc. Inf. Syst. AIS Electron. Libr.

[B92] Wu Y. Gao S. Ji B. Liu Z. Zeng X. Yu Z. (2020). Occurrence of short- and medium-chain chlorinated paraffins in soils and sediments from Dongguan City, South China. Environ. Pollut. 265, 114181. 10.1016/j.envpol.2020.114181 32806426

[B93] Wu Y. Wu J. Wu Z. Zhou J. Zhou L. Lu Y. (2021). Groundwater contaminated with short-chain chlorinated paraffins and microbial responses. Water Res. 204, 117605. 10.1016/j.watres.2021.117605 34488140

[B94] Xia D. Vaye O. Lu R. Sun Y. (2021). Resolving mass fractions and congener group patterns of C8−C17 chlorinated paraffins in commercial products: associations with source characterization. Sci. Total Environ. 769, 144701. 10.1016/j.scitotenv.2020.144701 33736236

[B95] Xu C. Wang K. Gao L. Zheng M. Li J. Zhang L. (2021). Highly elevated levels, infant dietary exposure and health risks of medium-chain chlorinated paraffins in breast milk from China: comparison with short-chain chlorinated paraffins. Environ. Pollut. 279, 116922. 10.1016/j.envpol.2021.116922 33743436

[B96] Yan L. Yao X. Zhang T. Jiang F. Shui Y. Xie H. (2023). Passivation effect of the chlorinated paraffin added in the cutting fluid on the surface corrosion resistance of the stainless steel. Molecules 28 (9), 3648. 10.3390/molecules28093648 37175058 PMC10180265

[B97] Yang L. Liu Y. Cui Z. Zhang Y. Zhang J. Lian K. (2021). Metabolomic mechanisms of short chain chlorinated paraffins toxicity in rats. Environ. Res. 197, 111060. 10.1016/j.envres.2021.111060 33798518

[B98] Yuan B. Haug L. S. Tay J. H. Padilla-Sánchez J. A. Papadopoulou E. de Wit C. A. (2022a). Dietary intake contributed the most to chlorinated paraffin body burden in a Norwegian cohort. Environ. Sci. and Technol. 56 (23), 17080–17089. 10.1021/acs.est.2c04998 36378808 PMC9730849

[B99] Yuan B. Hui Tay J. de Wit C. A. Papadopoulou E. Padilla-Sánchez J. A. Småstuen Haug L. (2023). Human exposure to chlorinated paraffins in scandinavia: association between human biomonitoring and external exposures to SCCPs, MCCPs, and LCCPs in a Norwegian cohort. Oslo Norway: Nordic Council of Ministers.

[B100] Yuan B. Rüdel H. de Wit C. A. Koschorreck J. (2022b). Identifying emerging environmental concerns from long-chain chlorinated paraffins towards German ecosystems. J. Hazard. Mater. 424, 127607. 10.1016/j.jhazmat.2021.127607 34768030

[B101] Yuan B. Tay J. H. Padilla-Sánchez J. A. Papadopoulou E. Haug L. S. de Wit C. A. (2021b). Human exposure to chlorinated paraffins via inhalation and dust ingestion in a Norwegian cohort. Environ. Sci. and Technol. 55 (2), 1145–1154. 10.1021/acs.est.0c05891 33400865 PMC7880561

[B102] Yuan S. Wang M. Lv B. Wang J. (2021a). Transformation pathways of chlorinated paraffins relevant for remediation: a mini-review. Environ. Sci. Pollut. Res. 28 (8), 9020–9028. 10.1007/s11356-021-12469-w 33475920

[B103] Zhang R. Li J. Wang Y. Jiang G. (2023). Distribution and exposure risk assessment of chlorinated paraffins and novel brominated flame retardants in toys. J. Hazard. Mater. 447, 130789. 10.1016/j.jhazmat.2023.130789 36641847

[B104] Zhang S. Horrocks A. R. (2003). A review of flame retardant polypropylene fibres. Prog. Polym. Sci. 28 (11), 1517–1538. 10.1016/j.progpolymsci.2003.09.001

[B105] Zhou Y. Yuan B. Nyberg E. Yin G. Bignert A. Glynn A. (2020). Chlorinated paraffins in human milk from urban sites in China, Sweden, and Norway. Environ. Sci. and Technol. 54 (7), 4356–4366. 10.1021/acs.est.9b06089 32101003 PMC7343287

[B106] Zhu C. Liu S. Cao Z. Hu B. Yang C. Luo X. (2024). Human dermal exposure to short- and medium-chain chlorinated paraffins: effect of populations, activities, gender, and haze pollution. J. Hazard. Mater. 476, 135169. 10.1016/j.jhazmat.2024.135169 39024769

